# Development and validation of a real-time PCR assay to detect *Cannabis sativa* in food

**DOI:** 10.1038/s41598-021-83908-4

**Published:** 2021-02-26

**Authors:** Sandra Weck, Verena Peterseil, Helmut K. Mayer, Rupert Hochegger

**Affiliations:** 1 Department for Molecular Biology and Microbiology, Institute for Food Safety Vienna, Austrian Agency for Health and Food Safety, Spargelfeldstrasse 191, 1220 Vienna, Austria; 2 Institute of Food Science, BOKU – University of Natural Resources and Life Science, Muthgasse11/1, 1190 Vienna, Austria

**Keywords:** Biochemistry, Genetics, Molecular biology, Plant sciences

## Abstract

Regarding the prospective investigation of food authenticity and adulteration the aim of the present study was the development and validation of a real-time PCR assay to identify hemp (*Cannabis sativa*) which has gained increasing importance as a valuable food ingredient. The assay targets a specific spacer DNA sequence in *Cannabis sativa* chloroplasts and detects 1.5 pg hemp DNA, which is equivalent to 18 copies/µL. Corresponding to the very low LOD (0.00031 ng/µL) the method allows the detection of hemp even in the infinitesimal concentration of contaminants. Due to a SNP in position 603, hemp can be identified unequivocally and discriminated from its closest relative hops (*Humulus lupulus)*. The PCR method shows no cross-reactivity with 39 of 46 tested plant species. Low cross-reactivity with mulberry, stinging nettle, lavender, cornflower, wine, figs and hops can be neglected, because the Δ Ct-values are > 14, and the obtained Ct-values are beyond the cut-off for a positive assessment (Ct-values ≤ 33). Moreover, the suitability of the method to identify hemp as a food ingredient was proved by analysing diverse food products such as chocolate or cookies.

## Introduction

In recent times, hemp has gained increased importance as valuable food ingredient regarding its beneficial macro- and microcomponent profile, and the necessity of sustainable plant sources caused by the growing population^1^. At present, eligible fibre hemp cultivars bred in Europe (*Cannabis sativa L*.) are permitted to exhibit a maximum THC (Tetrahydrocannabinol) content of 0.2% as determined by European Parliament´s and Council´s regulation no. 1307/2013^2^. Indeed, the cultivation of hemp was prohibited in several Western European countries in the middle of the twentieth century, because of its psychotropic potential^3^. However, in the beginning of the twenty-first century, the interest in the crop revived; at first merely regarded as a profitable by-product^4^, and subsequently marketed at health food shops as a niche product, valuable for human nutrition^3^. The high nutritional quality of hempseeds results from a well-balanced composition of macronutrients, consisting of 25–35% oil, 20–30% carbohydrates, 20–25% high digestible protein, and 10–15% insoluble fibre^5–7^. Moreover, they comprise noteworthy levels of minerals and vitamins, such as Vitamin E, A and C^8^. To further illustrate the quality of hempseeds, light shall be shed on the perfectly balanced fatty acid composition of hempseed oil^9^. Therefore, Montserrant-de la Paz examined the fatty acid profile of hempseeds by gas chromatography. They indicated that 76% of the total fatty acids are polyunsaturated, and 12% each monounsaturated and saturated. Thus, 88% of the total fatty acids are unsaturated; linoleic (55%), α-linolenic (16%), and oleic acid (11%) are the most abundant^6^. In addition, Vonapartis et al. confirmed the high content of unsaturated fatty acids, by examining the fatty acid profile of ten hemp cultivars with capillary gas chromatography and specified that they contain on average 90% unsaturated fatty acids. Accordingly, they verified that the abundant unsaturated fatty acids are linoleic acid (56.07%) and α-linolenic acid (15.98%), and that the major monounsaturated fatty acid is oleic acid (11.76%)^10^. As a result, the worth of the fatty acid profile of hempseed oil is determined by the essential fatty acids linoleic and α-linolenic acids, resulting in the favourable 3:1 ratio^5,6,11,12^, which was further evidenced by Vanapartis et al.^10^. In conclusion, the high amount of α-linolenic acid, the high ratio of polyunsaturated/saturated fatty acids, and further the ratio of ω-6/ω-3 fatty acids may have beneficial physiological effects on human nutrition. Besides, Multari et al. validated that also hemp in the form of flour has a valuable macro- and microcomponent profile, with a significant protein content of 38.55 ± 0.32% (w/w) and, moreover, is a rich source of insoluble fibre (25.49 ± 1.45%(w/w))^1^. In summary, hemp is a natural source of vegetable protein, containing all essential amino acids, with a simultaneous absence of inhibiting factors, which is an advantage over soy in vegetable-based and vegan nutrition. Similarly, hemp seeds are used to produce non-dairy products, such as desserts, bakery products or vegetable milk drinks and thus replace products made from soy, which conversely must be imported^13^ and is predominantly genetically modified. As a result, hemp is a sustainable and purely vegetable resource of protein, dietary fibre and valuable fatty acids, usable as a gluten free ingredient to process functional, healthy and less allergenic products, to satisfy environmentally and nutritionally conscious consumer´s demands. Consequently, more food producers might utilize hemp as an ingredient in their products and make an effort, to indicate the various beneficial physiological effects on human nutrition. As the interest in investigating the potential use of hemp in food products has increased in recent years^14^ the aim of the present study was the development and validation of a specific real-time PCR method that allows the precise detection of infinitesimal hemp traces, to respond to hemp as a current and prospective food ingredient. In terms of food safety, it is important to have valid methods available to verify and guarantee the food authenticity in the sense of consumer protection. So far, there are no extensively validated PCR methods available, which are used in routine analysis for the reliable detection of hemp and to ensure the authenticity of those food products. With regard to the prospective assessment of food authenticity and adulteration, this approach represents novelty in the area of food analytics.

## Results

### Variability

The sequence of the amplicon was compared to the DNA sequences available in the National Centre for Biotechnology Information (NCBI) sequence database using the Basic Local Alignment Search Tool (BLAST). As a result, the comparison showed 100% identity between the amplicon and the *Cannabis sativa* (AY958396) chloroplast genome. However, the identity between the amplicon and the chloroplast genome of hops (*Humulus lupulus)* (AB033890) was only 97%. Therefore, the gene region amplified with the primer pair Hemp_19Fw/Rv is suitable to distinguish hemp from hops.

**Figure 1:**
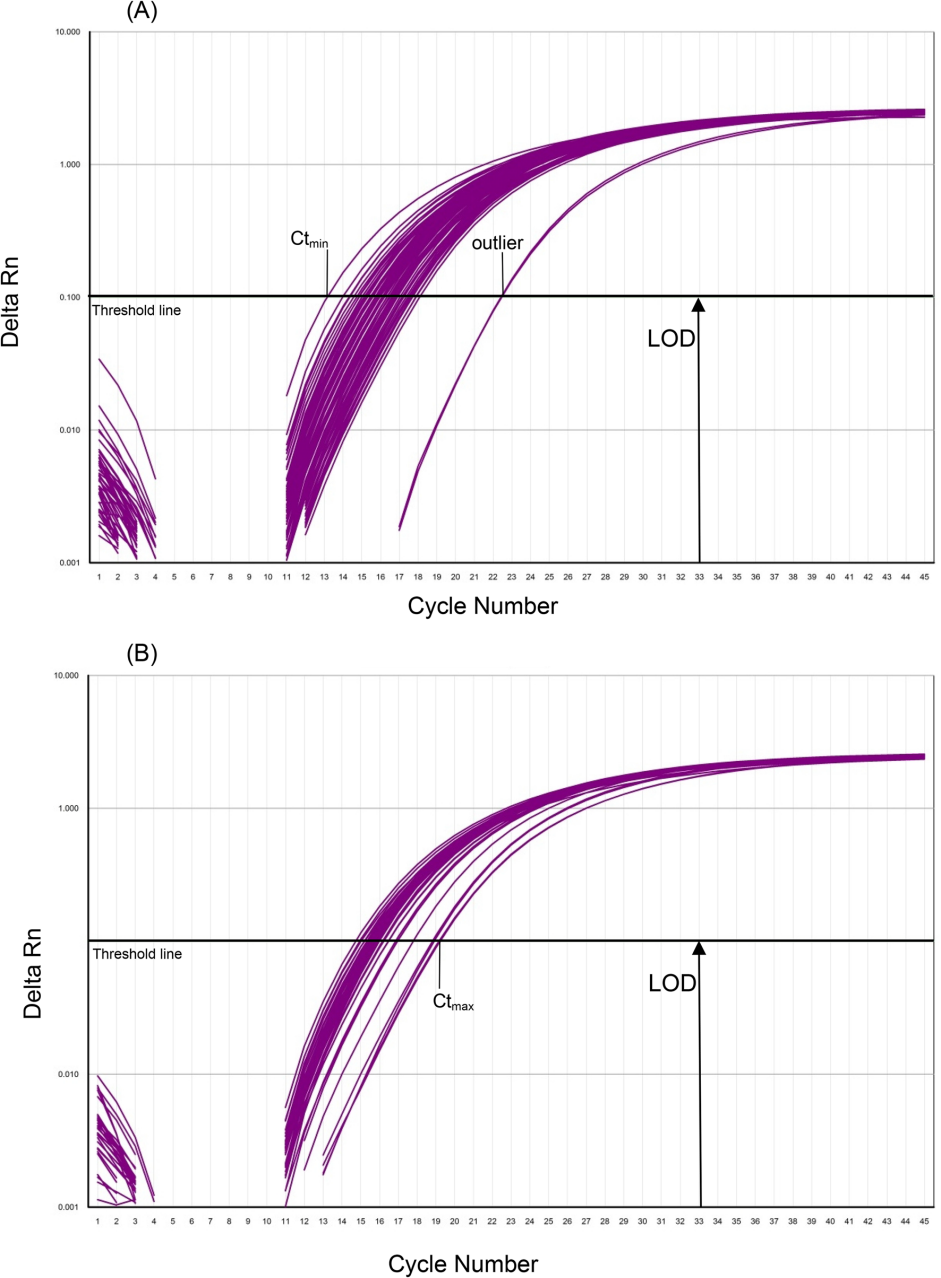

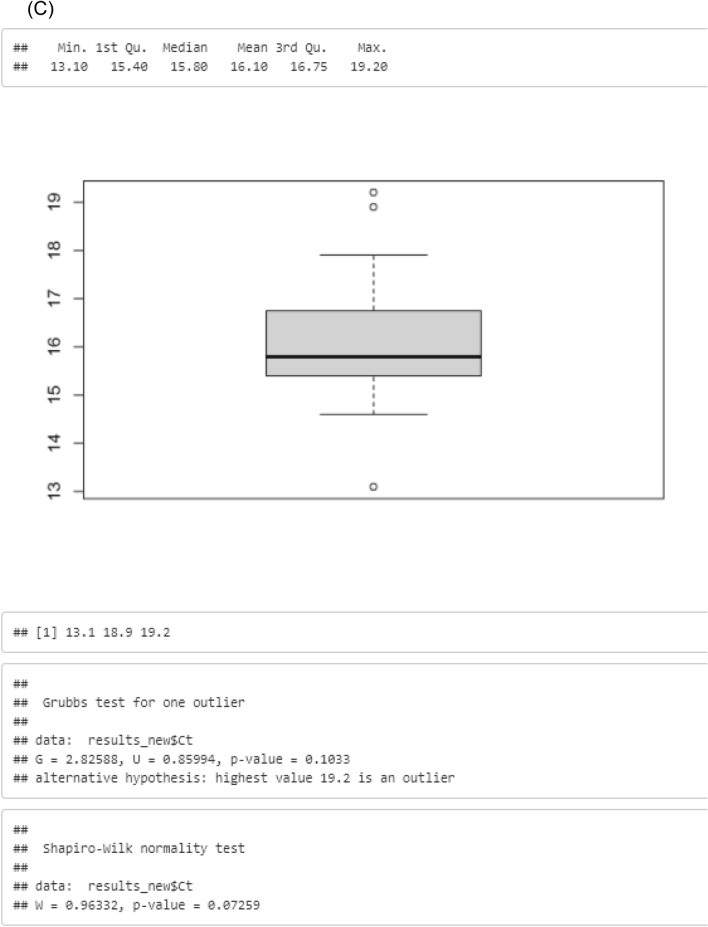
Intraspecific variability of hemp varieties with the numbers 1 and 21–101 (**A**) and with the numbers 4–20 and 107–128 (**B**) according to Table 1, obtained with 5 ng/µl DNA per tube. Results of Grubbs outlier and Shapiro-Wilks test (**C**).

The intraspecific variability indicates the variation that occurs within a species, and is influenced by genetic and environmental factors. The genetic diversity within *Cannabis sativa* was established by comparing 59 different varieties, using the previously described real-time PCR method with a DNA concentration of 5 ng/µL (Fig. 1a,b). To calculate the delta Ct—value, the minimum Ct-value, obtained with variety *Marcello*, was deducted from the maximum Ct-value, obtained with variety *Wojko*. As a result, the ∆Ct-value was calculated as follows: $\Delta \mathrm{Ct}-\mathrm{value }= {Ct}_{max}- {Ct}_{min }= 19.2 - 13.1 = 6.1$ (Table 1). Nevertheless, as can be shown it does not affect the detection per se. To further ensure that the obtained Ct values do not contain outliers and the population of samples is normally distributed, the program R was used to apply the Grubbs and the Saphiro-Wilk test. At first, the Grubbs outlier test founds that the extreme value 22.4 (*Novosadska*) is significantly different from the other data, therefore it is viewed as an outlier (Table 2, Fig. 1c). To verify that the test statistics is not distorted, the test for normal distribution should be performed without the presumed outliers. However, the PCR analysis with the variety *Novosadska* was repeated in 3 double approaches (data non shown), to rule out that an error has occurred during the approach. By repeating the PCR analysis, a $\stackrel{-}{\mathrm{X}}$Ct value of 16.1, which is now in the range of the Ct values obtained by the remaining population, was obtained. Subsequently, the Grubbs test was applied one more time with new maximum value of 19.2 (*Wojko*). The Grubbs outlier test confirmed that the new extreme value 19.2 is not significantly different from the other data, i.e. it is not an outlier. The Grubbs test assumed normally distributed data. Furthermore, the test for normal distribution using the Shapiro-Wilks test showed that the null hypothesis of normal distribution can be maintained (Fig. 1c). To sum up, we were able to proof, that the Ct values are normally distributed and do not differ significantly from one another^15^.

**Table 1:** Analysis of hemp varieties obtained with pool samples consisting of 5 seeds each.

Variety denomination	X̅ Ct-value of duplicate set-up	Sender of samples	Sample type
Anbo	16.7	Agricultural Institute of Slovenia (Ljubljana)	S
Asso	18.9	Centro di Sperimentationee Certificazione delle Sementi (Milano)	S
Beniko	16.2	Main Inspectorate of Plant Health and Seed Inspection (COBORU)	S
Bialobrzeskie	15.8	Institute of Natural Fibres and Medicinal Plants	S
Campangnola	16.9	Hanfland (Laa/Thaya)	C
Carmaleonte	16.1	Centro di Sperimentationee Certificazione delle Sementi (Milano)	S
Carmagnola	17.4	Centro di Sperimentationee Certificazione delle Sementi (Milano)	S
Chamaeleon	16.0	NAK (Emmeloord)	S
Codimono	15.8	Centro di Sperimentationee Certificazione delle Sementi (Milano)	S
CS	17.2	Centro di Sperimentationee Certificazione delle Sementi (Milano)	
Dioica 88	16.1	G.I.P.- G.E.V.E.S. (Surgeres)	S
Eletta Campana	15.4	Centro di Sperimentationee Certificazione delle Sementi (Milano)	S
Epsilon 68	15.3	G.I.P.- G.E.V.E.S. (Surgeres)	
Fedora 17	16.7	G.I.P.- G.E.V.E.S. (Surgeres)	S
Felina 32	14.6	G.I.P.- G.E.V.E.S. (Surgeres)	S
Fedora 19	15.4	G.I.P.- G.E.V.E.S. (Surgeres)	S
Fedrina 74	14.9	G.I.P.- G.E.V.E.S. (Surgeres)	S
Ferimon	16.6	G.I.P.- G.E.V.E.S. (Surgeres)	S
Fibranova	17.8	Centro di Sperimentationee Certificazione delle Sementi (Milano)	S
Fibrante	16.2	Centro di Sperimentationee Certificazione delle Sementi (Milano)	
Ferimon 12	16.2	FNPC	M
Fibrimor	15.4	Centro di Sperimentationee Certificazione delle Sementi (Milano)	S
Fibrimon 56	17.7	G.I.P.- G.E.V.E.S. (Surgeres)	S
Fibrol	16.4	Agromag KFT (Szeged)	M
Finola	15.6	NAK (Emmeloord)	S
Futura	15.0	G.I.P.- G.E.V.E.S. (Surgeres)	S
Futura 75	17.1	G.I.P.- G.E.V.E.S. (Surgeres)	S
Goricka simba	16.8	Agricultural Institute of Slovenia (Ljubljana)	S
Helena	17.9	Institute of Field and Vegetable Crops (Novi Sad)	S
Idalgo	17.4	Centro di Sperimentationee Certificazione delle Sementi (Milano)	
Ivory	16.8	NAK (Emmeloord)	S
JTF	15.2	Hanfland (Laa/Thaya)	C
KC Dora	15.7	Agromag KFT (Szeged)	M
KC Virtus	17.8	Nebih	S
KC Zuzana	15.7	Agromag KFT (Szeged)	M
Kompolti	15.8	NAK (Emmeloord)	S
Kompolti hibrid TC	15.7	Nebih	S
Kompolti NRG	16.2	Nebih	S
Kompolti kender	15.2	Nebih	S
Lipko	15.2	Nebih	S
Lovrin 110	16.3	P.H. Petersen (Lundsgaard)	
Maja	17.5	Agricultural Institute of Slovenia (Ljubljana)	S
Marcello	13.1 = Ct_min_	NAK (Emmeloord)	S
Markant	17.2	NAK (Emmeloord)	S
Monoica	14.8	UKZUZ (TCH)	S
Novosadska	*(22.4)* = *outlier*	Institute of Field and Vegetable Crops (Novi Sad)	S
	16.1		
Santhica 23	14.6	G.I.P.- G.E.V.E.S. (Surgeres)	
Santhica 27	15.6	G.I.P.- G.E.V.E.S. (Surgeres)	S
Santhica 70	14.8	G.I.P.- G.E.V.E.S. (Surgeres)	S
Santhica morcna	15.5	Agritec (Sumperk)	CE
Silesia	15.4	Main Inspectorate of Plant Health and Seed Inspection	S
Szarvasi	16.4	Nebih	S
Tiborszallasi	15.5	Centro di Sperimentationee Certificazione delle Sementi (Milano)	S
Tisza	15.4	Agromag KFT (Szeged)	M
Tygra	15.6	Institute of Natural Fibres and Medicinal Plants	S
Uniko B	15.1	Nebih	S
USO-31	15.8	NAK (Emmeloord)	S
Wielkopolskie	15.2	Main Inspectorate of Plant Health and Seed Inspection	S
Wojko	19.2 = Ct_max_	Institute of Natural Fibres and Medicinal Plants	S
Average Ct-value X̅^a^	16.1		
Standard deviation STD^b^	1.09		
∆ Ct-value^c^	6.10		

C: Consumer products/Commodities, for the purpose of further processing e.g. for feed production

CE: Certified material in the approval process, which is not yet admitted

M: Breeder cultivates the samples for the purpose of maintenance of the variety on behalf of the Owner

S: Standard samples from national Community Plant Variety Office

^a^$$\overline{{\text{X }}} = { }\frac{{x_{1} + x_{2} + \cdots + x_{n} }}{n}.$$

^b^$$STD{ } = \sqrt {\mathop \sum \limits_{i = 1}^{n} \frac{{\left( {xi - {\upmu }} \right)^{2} }}{n}} .$$

^c^$$\Delta {\text{C}} - {\text{value }} = Ct_{max} - { }Ct_{min} .$$

**Table 2:** Analysed food samples.

Name	Supplier	Brand	Composition	Hemp content
Hemp patty	Drugstore	hanf&natur	Chickpeas, 10% peeled hemp seeds, parsley, rock salt, hemp flour 4%, onions, spices, tartar	14%
Nibble hemp seeds	Drugstore	hanf&natur	Roasted hempseeds, whole cane sugar, vanilla sugar, cinnamon	~ 100%
Hemp cookies with chocolate	Sponsoring	hanf&natur	Wheat flour, palm fat, cane sugar, 7.5% hemp flour, peeled hemp seeds, chocolate drops, cacao, pure tartar powder, sea salt	15%
Hemp cookies	Drugstore	hanf&natur	Spelt whole grain flour, palm fat, agave syrup, 7% peeled hemp seeds, 2% hemp flour, spices, tartar baking powder, rock salt	9%
Hemp-spelt pasta	Sponsoring	hanf&natur	95% spelt flour, 5% hemp flour	5%
Hemp spaghetti	Drugstore	hanf&natur	Semolina, 12% hemp flour	12%
Chocolate (dark)	Drugstore	hanf&natur	Cacao mass, cane sugar, 12% hemp seeds, cacao butter (cacao min. 70%)	12%
Chocolate (whole milk)	Sponsoring	hanf&natur	Cane sugar, cacao butter, whole milk powder, 12% hemp seeds, cacao mass, cream powder, skim milk powder, bourbon vanilla extract	12%
Chocolate (white)	Sponsoring	hanf&natur	Cane sugar, cacao butter, whole milk powder,10% spiced hemp seeds (hemp seeds, cane sugar, chili, curry, paprika, pepper, salt), skim mild yoghurt powder, bourbon vanilla extract	10%
Hemp pesto (green)	Drugstore	hanf&natur	Germinated sunflower seeds, rape kernel oil, garlic oil macerate, 8% peeled and germinated hemp seeds, dried tomatoes, dried basil, wild garlic salt	8%
Hemp flour	Drugstore	hanf&natur	Hemp seeds	100%
Hemp fruit bar	Sponsoring	hanf&natur	Sultanas, dates, honey marzipan, apricot, hazelnut, roasted hemp seeds 10%, 4-grain flakes (oat, wheat, rye, barley), banana, roasted sesame, vanilla, wheat wafers	12%
Hemp tea	Drugstore	hanf&natur	Dried hemp leaves, stinging nettle, blackberry leaves	Unknown
Hemp-nut muesli	Drugstore	hanf&natur	Barley flakes, whole spelt flakes, whole oat flakes, whole corn flakes, sunflower seeds, peeled hemp seeds, hazelnuts, linseed	~ 12%

### Validation of the real-time PCR analysis

#### Limit of detection (LOD) and range of linearity

**Figure 2:**
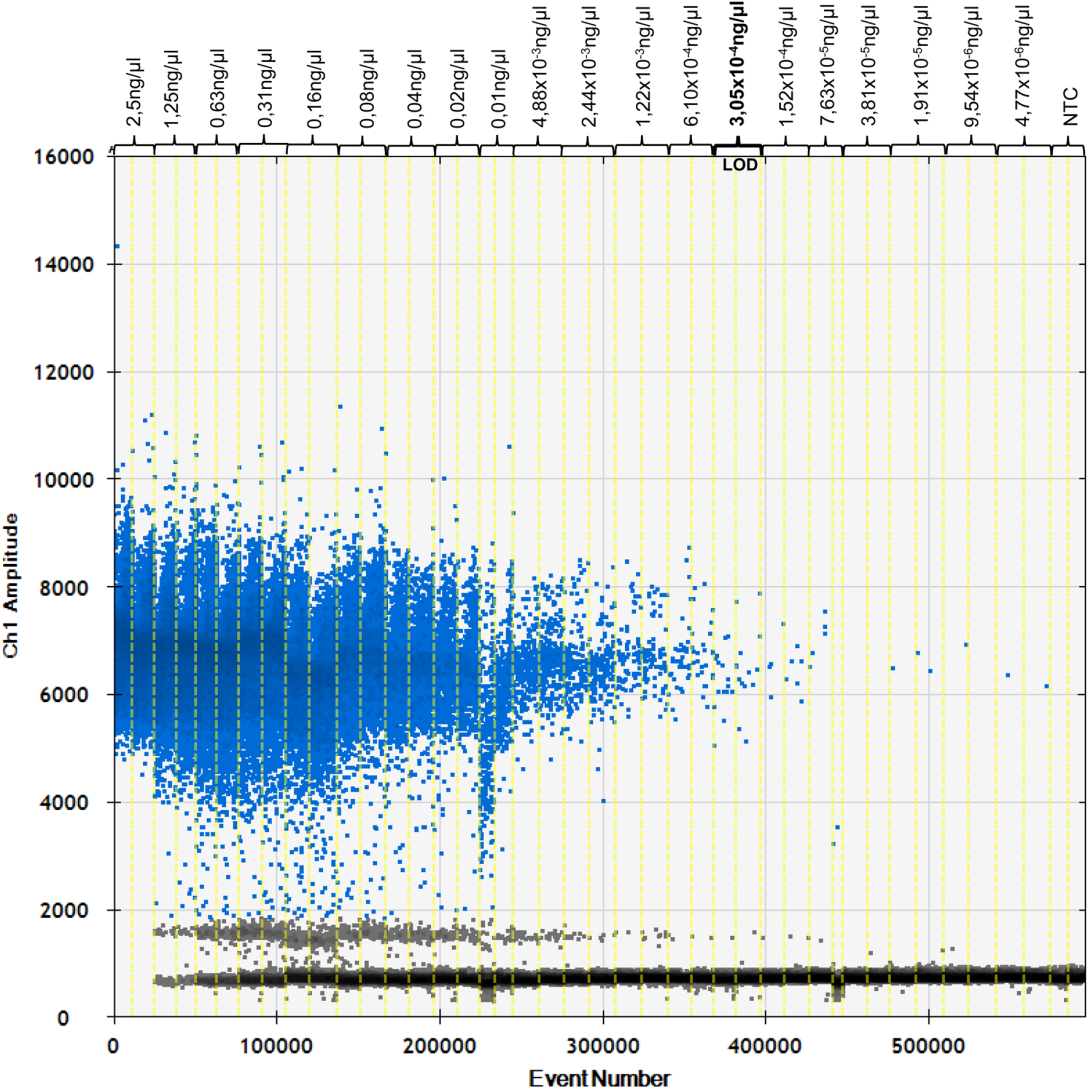
Results of determination of limit of detection (LOD) with digital droplet PCR, using DNA extracts from *Novosadska* seeds in concentrations from 2.5 ng/µL to 4.77 × 10–6 ng/µL.

The limit of detection was established by analysing serially diluted DNA extracted from *Novosadska* seeds with real-time PCR in concentrations from 2.5 ng/µL to 4.77 × 10^–6^ ng/µL, corresponding to a total DNA amount from 25 ng to 0.024 pg; the limit of detection was set at 0.00031 ng/µL, which is equivalent to a Ct-value of 32 ± 1. As a result, an average Ct-value of 32 ± 1 could be assigned as cut-off to evaluate a positive or negative result; thus, an increase of the fluorescence signal within 33 cycles was considered as a positive result. To calculate the range of linearity, the average Ct-value was plotted against the logarithmic DNA concentration of the corresponding dilution stage (R^2^ = 0.9975). In addition, serially diluted DNA extracts from *Novosadska* seeds were analysed with digital droplet PCR in concentrations from 2.5 ng/µL to 4.77 × 10^–6^ ng/µL, to determine the number of copies, corresponding to the respective dilution levels (Fig. 2). As a result, the determined limit of detection is equivalent to 358 copies/20 µL or 18 copies/µL. By analysing samples in 10 replicates, the certain amplification near the limit of detection could be evidenced with real-time PCR as well as digital droplet PCR.

#### Selectivity

In this context, selectivity means that the target DNA can be determined reliably, even in the presence of potentially disturbing non-target DNA. Therefore, identifying at least 1% target DNA was defined as the minimum requirement. The defined minimum requirement was accomplished; even much less target DNA concentrations are verifiable. As a result, the method proofs selectivity over each analysed concentration from 50 ng/µL to 4.77 × 10^–5^ ng/µL, which corresponds to an amount of 90.9% to 0.00048% target DNA (data non shown).

#### Robustness

The robustness of a PCR detection method is an indication of the method’s reliability during normal usage, and permits certain variation in method parameters, including the temperature-time protocol as well as the DNA amount and quality, but nevertheless provides reproducible results of the same value.

The previously defined minimum requirement was accomplished regarding all simulated pipetting errors with an initial annealing temperature of 61 °C and a decreased annealing temperature of 60 °C. Furthermore, the minimum requirement was accomplished using a different thermocycler (Rotor-Gene Q, Promega, Netherlands) and an annealing temperature of 61 °C. Merely, increasing the annealing temperature to 62 °C, results in Δ Ct-values exceeding 1. In conclusion, the robustness of the method was approved for using increased or reduced DNA quantity (± 1 µl), provided that an annealing temperature between 61 and 60 °C is applied (data non shown).

#### Specificity

**Figure 3:**
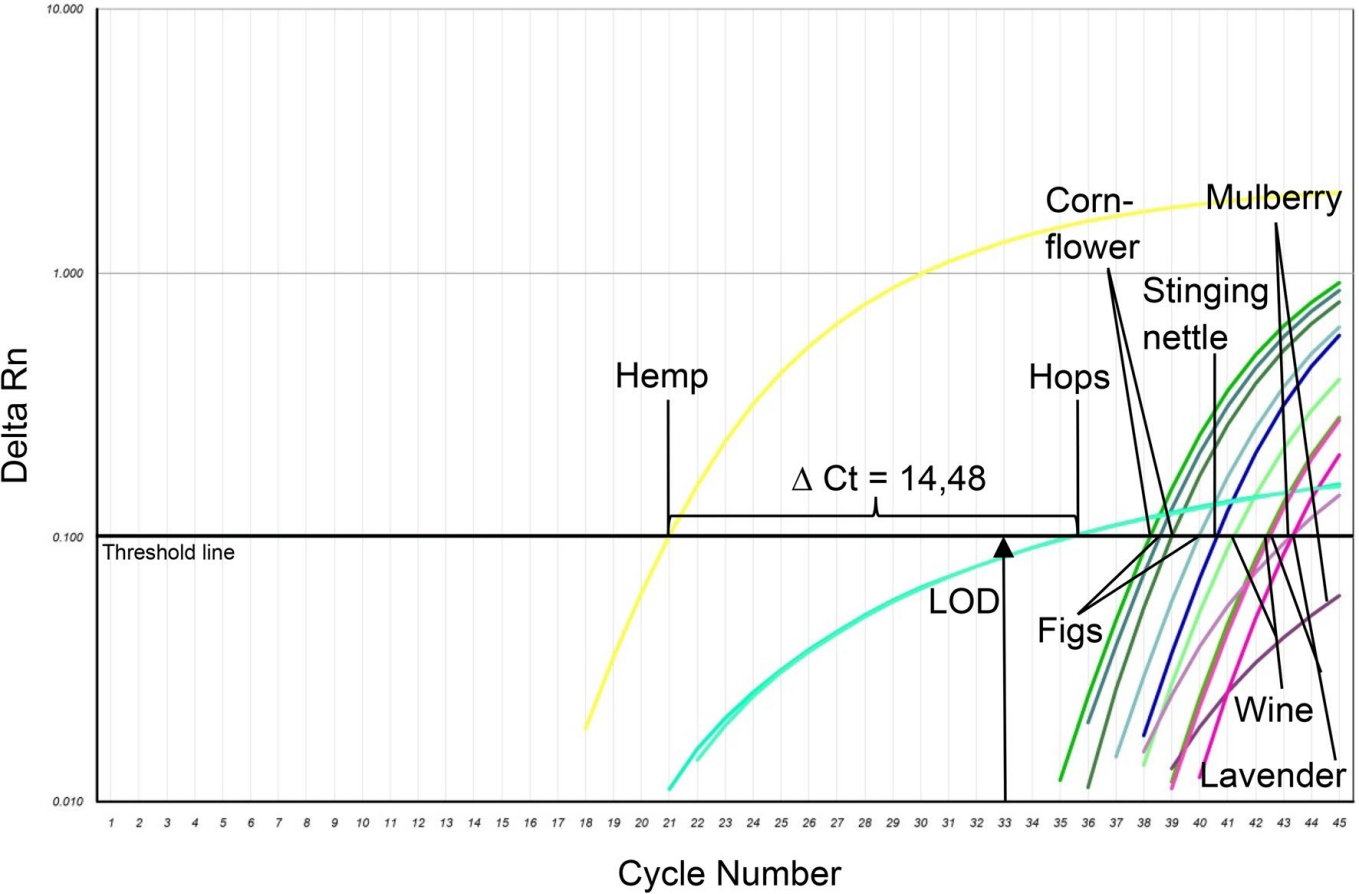
Results of cross-reactivity tests obtained with 5 ng/µl DNA per tube. Cross-reacting species: mulberry, stinging nettle, lavender, cornflower, wine, figs and hops. The cross-reactivity is negligible, because the ∆ Ct values are > 14 and the Ct values are beyond the determined limit of detection.

The PCR method is considered as specific if it is capable of detecting and amplifying the appropriate DNA sequence exclusively. As a result, the presented real-time PCR method did not show cross-reactivity with 39 tested species. However, PCR analyses of DNA extracts from mulberry, stinging nettle, lavender, cornflower, wine, figs and hops leaves, showed amplification curves with Δ Ct-values > 14 in comparison with hemp (Fig. 3). The obtained Ct-values are sufficiently high to enable a clear distinction, wherefore the cross-reactivities are negligible. Moreover, the obtained Ct-values were beyond the cut-off for a positive evaluation (Ct-values ≤ 33), which is equivalent to a DNA concentration of 0.00031 ng/µl. Furthermore, due to differences in the reaction kinetics, the amplification curve of hops visually differed significantly from the curve provided by hemp. Consequently, the method is suitable to distinguish the analysed species from hemp within the limit of detection.

### Analysis of food samples

**Figure 4:**
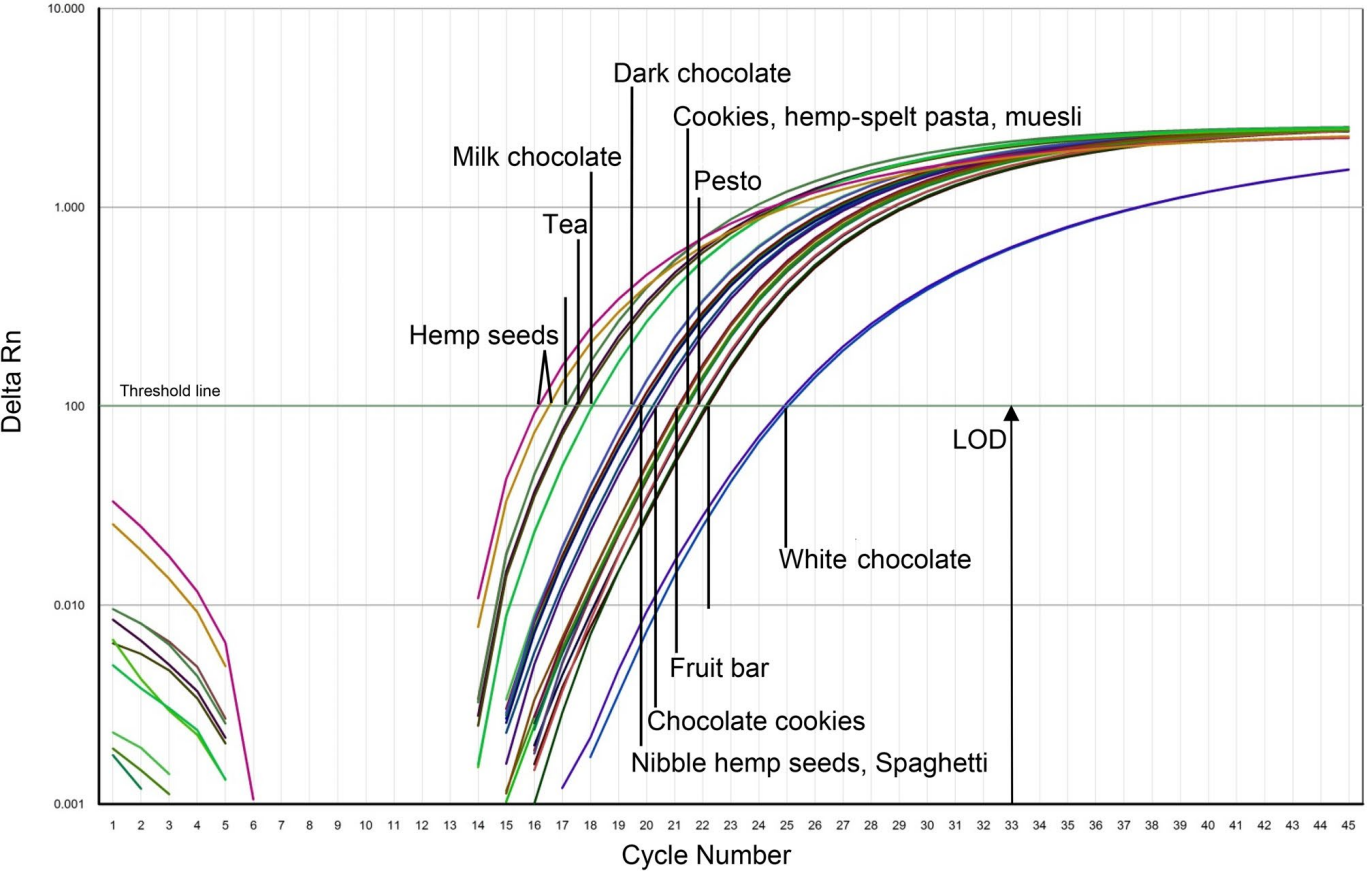
Results of analysing diverse foodstuff obtained with 5 ng/µl DNA per tube. Hempseeds were analysed as reference material**.**

The suitability of the developed real-time PCR method to identify hemp as a food ingredient could be evidenced by analysing diverse composed food products in comparison to hemp seeds (Fig. 4). DNA extracts from white chocolate showed the highest Δ Ct-values in comparison to hemp seeds (Δ Ct = 8.68), whereas the extracts from tea (Δ Ct = 0.81) and flour (Δ Ct = 1.16) showed minimal differences in the Ct-values. Most food samples provided Δ Ct-values between 3.13 and 5.83 compared to hemp seed. As can be expected, less or untreated food products, such as tea (Δ Ct = 0.81) and hemp flour (Δ Ct = 1.16), show Ct-values close to that of hemp seeds, which have been used during method development and validation. Due to the low degree of processing, the DNA has a comparably good quality status. Nevertheless, during manufacturing processes such as grinding, drying, or baking, the DNA is increasingly destroyed, which reduces both quantity and quality of amplifiable DNA. Moreover, interfering ingredients such as sugar or fat have a decisive influence on the DNA quality and quantity during extraction. From this fact one can deduce that with an increasing degree of processing and composition, the amplification curves will rise later and thus the Ct values will be higher compared to hemp seeds. This is confirmed by the results obtained by analysing chocolate, fruit bar or pesto (Fig. 4). Especially white chocolate which mainly consists of cane sugar and cacao butter has a striking hight Ct- value (Δ Ct = 8.68), what indicates a low presence of amplifiable DNA.

## Discussion

The real-time PCR method is based on the detection of a spacer DNA sequence between *the trnL* 3´exon and the *trnF* gene in *Cannabis sativa* chloroplasts, which was first identified by Linacre and Thorpe in 1998^16^. The present assay is specific for hemp and appropriate to identify 1.5 pg hemp DNA, which is equivalent to 18 copies/µL. The real-time PCR method does not show cross-reactivity with 39 of 46 tested herbs, spices, nuts and cereals frequently used as food ingredients. However, it shows slight cross-reactivity with mulberry, stinging nettle, lavender, cornflower, wine, figs and hops. Nevertheless, the cross-reactivity can be neglected, because the Δ Ct-values are > 14, which is sufficiently high to enable a clear distinction, and the obtained Ct-values are beyond the determined cut-off for a positive assessment (Ct-values 33 ≙ 0.00031 ng/µl). Consequently, the method is suitable to identify hemp unequivocally and to distinguish hemp from the analysed species, including its closest relative hops (*Humulus lupulus).* In contrast to other current studies, examining the DNA-based identification of *Cannabis sativa,* the present assay convinces with more comprehensive specificity tests. For example, Jeangkhwoa et al. performed cross-reactivity tests with four^17^, Kitamura et al. with seven^18^, Houston et al. with fourteen plant species^19^ and Johnson et al. with hops only^20^, whereas the present study covers 46 relevant plant species. Moreover, the method remains almost unaffected by increasing and reducing the DNA quantity by 1 µL, provided that an annealing temperature between 61 °C and 60 °C is applied (Δ Ct-values < 0.5). However, increasing the annealing temperature to 62 °C results in major Δ Ct-values > 1.8 compared to normal usage at 60 °C. To evaluate the reproducibility of the method, further analyses in different laboratories and conducted by different operators are necessary. Furthermore, 59 hemp varieties from 9 countries were assessed with the real-time PCR method described in the present paper to establish the variation that occurs within *Cannabis sativa*. As a result, the calculated delta Ct- value (∆ Ct = 6.1) presents the intraspecific variability of the analysed hemp varieties. These results support the findings from Soler et al., who observed a high polymorphism by genotyping 22 *Cannabis sativa* cultivars using *genomic Simple Sequence Repeat (gSSRs)*^21^, although they examined different gene sequences*.* However, to obtain an adequate overview of the intraspecific variability, that analysis of a few specimens is insufficient. The discovered intraspecific variability is neither surprising nor disturbing, but rather a result of varying environmental influences and climatic conditions but as can be shown, does not affect the detection of hemp per se. Furthermore, Cannabis sativa shows a high genetic diversity resulting also in minor variations on the target sequence which can lead to a lower PCR efficiency. To confirm these results and particularly the variation on the targeted gene sequence even more valid, sequencing and comparing different hemp varieties would be further important in the future. To get a first overview, sequencing and comparing those varieties with Ct_max_ (Wojko), Ct_min_ (Marcello) and Ct_X̅_ would be a suggestion. As it is not yet known how stable the investigated gene is, it would be necessary to analyse at least 10 individual grains per variety to obtain a first valid result.

The suitability of the method to identify hemp as a food ingredient could be confirmed by analysing diverse composed food products. In summary, all analysed food samples provide Δ Ct-values ≤ 8.68. It can be assumed that DNA extracts from less processed or compound food products, such as tea (Δ Ct = 0.81) and flour (Δ Ct = 1.16), show lower Δ Ct-values than food products with high sugar and fat content, such as white chocolate (Δ Ct = 8.68). Regarding the identification of hemp in food products, the present real-time PCR method represents novelty and meets the cutting-edge requirements of food analytics derived from Leitlinien zur Einzellabor-Validierung qualitativer real-time PCR Methoden^22^ and the MIQE Guidelines^23^, adapted to the publication of qualitative methods. Although several chemical (e.g., HPLC or GC) and biological approaches are suitable to detect the presence of THC in food^24^, the present paper provides a universally applicable and highly specific real-time method to unequivocally determine the ingredient hemp as species per se in food products, regardless of the presence or absence of THC. In comparison to the real-time PCR method published by Johnson et al.^20^ and the DNA-based method to identify hemp in pastry^25^, the present study convinces with a shorter amplicon length (122 bp), which is preferable concerning the analysis of complex food samples, with potentially highly degraded DNA, caused by the application of diverse manufacturing processes such as heating or fermenting.

## Conclusion

Based on the extensive and comprehensive validation of the real-time PCR assay, this approach represents novelty in the area of food analytics. As a result, the method serves as a highly accurate instrument to determine infinitesimal hemp traces down to 0.00031 ng/µL and is suitable to reveal adulteration and to ensure the authenticity of hemp food product. However, it enables qualitative analyses exclusively. With regard to the prospective assessment of food authenticity and adulteration, further research in the development of quantitative PCR methods is necessary.

## Materials and methods

### Acquisition of hemp varieties

To analyse the existing hemp varieties as completely as possible, the section of Seed and Propagating Material of the Austrian Agency for Health and Food Safety investigated hemp varieties in the National Variety List of the EU member states (currently 28 member states) as well as in the Common EU Catalogue of Varieties of Agricultural Plant Species, and requested available seed samples listed in Table 1. Subsequently, mainly standard samples provided by the national Community Plant Variety Office, but also hemp varieties from breeders and maintainers were analysed.

The food products were kindly provided by Hanf & Natur (http://www.hanf-natur.com/, Gerberstr.24, 51,789 Lindlar, Germany), and are acquirable in food retail trade as well as in drugstores and health food stores.

### Sample preparation and DNA extraction

#### Seed samples

Composite samples of 5 hempseeds were ground in a *Precellys* 2 ml Tube, contained in the grinding kit MK28 with 2.8 mm steel beads, using the *Minilys* Tissue Homogenizer (Bertin Technologies, France). The DNA was extracted with the *NucleoSpin Plant II Kit* (Macherey-Nagel, Germany). Based on the measured concentration, the extracts were diluted to an operating concentration of 5 ng/µL for subsequent PCR analysis.

#### Food and leaf samples

Food samples described in Table 2 were ground and homogenized using the Knife Mill *Grindomix GM200* (Retsch, Germany). Subsequently, the DNA was extracted according to the *CTAB* protocol using the *Maxwell 16 FFS Nucleic Acid Extraction System Custom-Kit* (Promega, USA). The chocolate was defatted with acetone and petroleum-benzine prior to the extraction. Leaf samples used for the cross-reactivity analyses were deep-frozen with liquid nitrogen and homogenized using a mortar. Subsequently, the DNA was extracted analogous to the food samples. To calculate the DNA concentration of the DNA extracts, the absorption at 260 nm was measured with a UV/Vis spectrometer (QIAxpert System, Qiagen, Netherlands). Based on the measured concentration, the extracts were diluted to an operating concentration of 5 ng/µL for subsequent PCR analysis.

### Primers and probes

**Figure 5:**
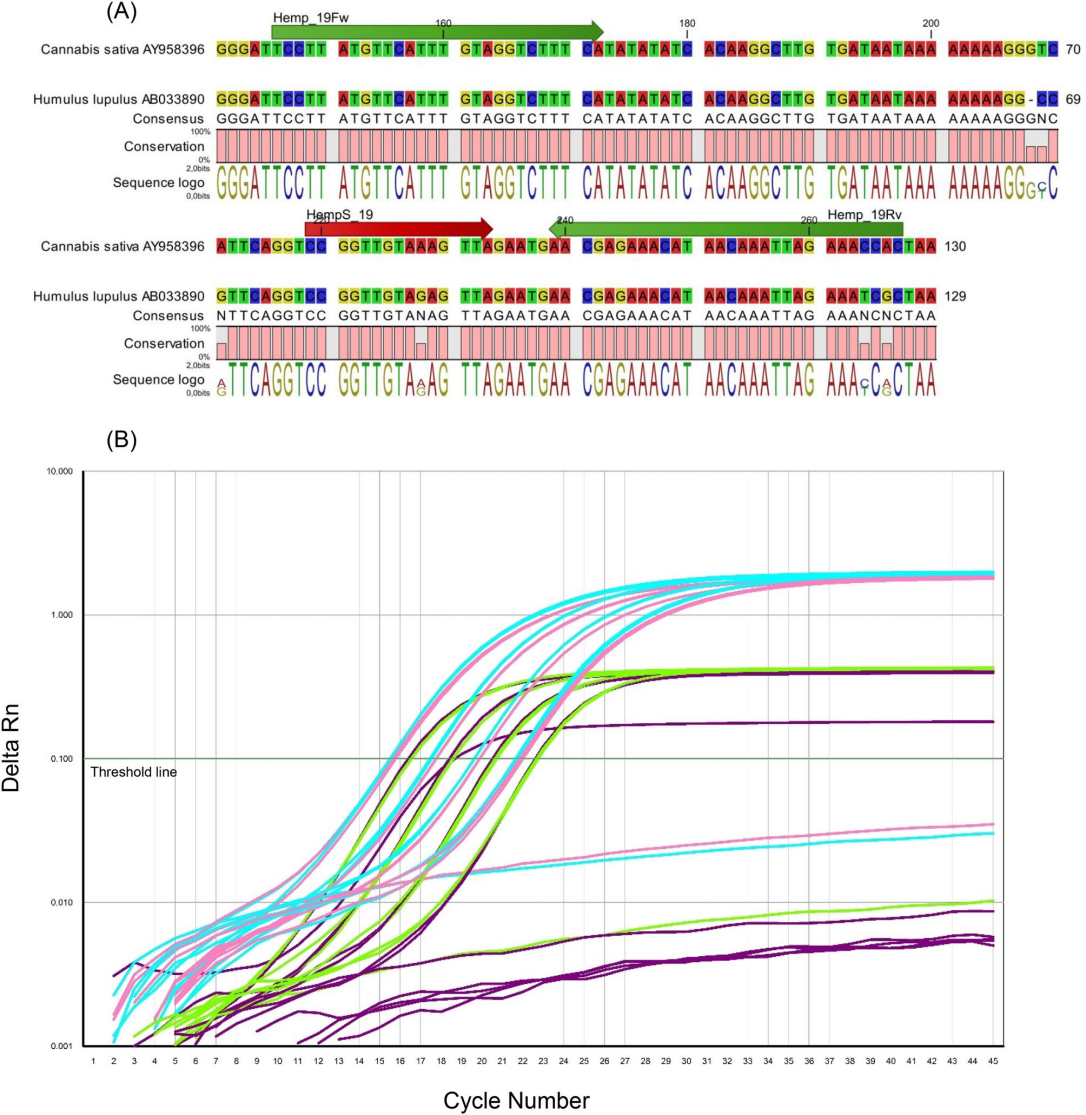
(**A**) Position of primers and probe presented in the spacer DNA sequence between the *trnL* 3´exon and the *trnF* gene in *Cannabis sativa* chloroplasts. The probe HempS_19, marked in red, discriminates hemp from hops based on a SNP in position 603. The primer pair Hemp_19 Fw/Rv is marked in green. (**B**) Amplification curves obtained with tested primer and probe concentrations by analysing *Monoica* seeds with DNA concentrations of 100 ng/µl, 25 ng/µl, 6.25 ng/µl and 1.5625 ng/µl. (1) Purple: 0.5 µmol/L per primer, 0.1 µmol/L probe. (2) Light blue: 0.5 µmol/L per primer, 0.25 µmol/L probe. (3) Rose: 0.25 µmol/L per primer, 0.25 µmol/L probe. (4) Green: 0.25 µmol/L per primer, 0.1 µmol/L probe.

In the beginning of the study, the following primer/probe set was designed, to target the hemp-specific spacer DNA sequence between the *trnL* 3´exon and the *trnF* gene in *Cannabis sativa* chloroplasts (Fig. 5a). Primers: Hemp_19Fw: TCC TTA TGT TCA TTT GTA GGT CTT TCA, Hemp_19Rv: GTG GTT TC TAA TTT GTT ATG TTT CTC GTT, Probe: HempS_19: **NED**-CCG GTT GTA AAG TTA-**MGBNFQ**. The amplicon length is 122 base pairs (bp). The optimal primer and probe concentrations (0.25 µmol/L per primer and 0.25 µmol/L probe) were determined by developing a primer/probe matrix; results can be seen in Fig. 5b. The concentration schemes 2 and 3 provide the most suitable amplification curves with optimal exponential and plateau phase (Fig. 5b). For practical reasons, concentration scheme 3, consisting of 0.25 µmol/L per primer and 0.25 µmol/L probe, was selected for the PCR analysis. The primers Hemp_19Fw, Hemp_19Rv and the probe HempS_19 were designed using the software *Primer Express 3.0* (Applied Biosystems, USA). The probe is labelled on the 5´end with the reporter dye NED (real-time PCR) or FAM (ddPCR) and on the 3´end with a minor groove binding non-fluorescent quencher (MGBNFQ). The optimal primer and probe concentrations (Fig. 5b; scheme 3) were determined by developing a primer/probe matrix combining the primer concentrations 0.5 µmol/L and 0.25 µmol/L with the probe concentrations of 0.1 µmol/L and 0.25 µmol/L, respectively.

### Real-time PCR

The real-time PCR reactions were performed in *MicroAmp Optical 96-Well Reaction Plates* (Applied Biosystems, USA) in a total reaction volume of 25 µL. Each assay comprises 12.5 µL *Universal Master Mix* (Applied Biosystems, USA), 0.06 µL probe as well as both primers, 7.31 µL in-house bi-distilled water and 5 µL DNA. The real-time PCR assays were conducted using the *ABI 7500 Real-Time PCR System* (Applied Biosystems, USA) and the following temperature-time protocol: 2 min at 50 °C, 10 min at 95 °C followed by 45 cycles of 15 s at 95 °C and 1 min at 61 °C. A suitable threshold value of 0.101 was specified by choosing the middle of the linear phase of the amplification graphs.

### Digital droplet PCR (ddPCR)

To determine the number of copies at the limit of detection, the ddPCR reactions were performed in semi-skirted, colourless, *twin.tec PCR Plates 96* (Eppendorf, Germany) in a total reaction volume of 20 µL. Each assay comprises 10 µL *ddPCR Supermix for Probes (No dUTP)* (Bio-Rad, USA), 0.05 µL probe as well as primers, 5.85 µL in-house bi-distilled water and 20 ng DNA (5 ng/µL). The ddPCR assays were conducted using the *QX200 Droplet Generator* as well as *QX200 Droplet Reader* (Bio-Rad, USA), *Mastercycler* (Eppendorf) and the following temperature-time protocol: 2 min at 50 °C, 10 min at 95 °C followed by 45 cycles of 15 s at 95 °C and 1 min at 61 °C.

### Experimental design

#### Variability

The sequence of the amplicon was compared to the DNA sequences available in the National Centre for Biotechnology Information (NCBI) sequence database using the *Basic Local Alignment Search Tool* (BLAST). To further determine the intraspecific variability, 59 hemp varieties (Table 1) were diluted to a concentration of 5 ng/µL and analysed with the previously described real-time PCR method. To ensure that the obtained Ct values do not contain outliers and the sample population is normally distributed, the program R was used to apply the Grubbs and the Saphiro Wilk test.

#### Limit of detection (LOD) and range of linearity

To establish the limit of detection, serially diluted DNA extracted from *Novosadska* seeds, were analysed with real-time PCR in concentrations from 2.5 ng/µL to 4.77 × 10^–6^ ng/µL. To calculate the range of linearity, the average Ct-value was plotted against the logarithmic DNA concentration of the corresponding dilution stage. In addition, serially diluted DNA extracts from *Novosadska* were analysed with digital droplet PCR in concentrations from 2.5 ng/µL to 4.77 × 10^–6^ ng/µL, so that the number of copies corresponding to the respective dilution levels could be determined. Finally, to evidence the certain amplification near the limit of detection, the samples were analysed in 10 replicates in the relevant concentration with real-time PCR as well as with digital droplet PCR.

#### Selectivity

To investigate the selectivity of the method, the hemp DNA extracts were serially diluted with non-target DNA with an initial concentration of 10 ng/µL. The non-target DNA was extracted from *Wisteria* leaves, belonging to the *Fabaceae* family. Consequently, the percentage of target DNA varies from 90.9 to 0.00048% concerning the total approach. The internally defined minimum requirement demands that at least 1% target DNA shall be identifiable. This requirement has proven to be effective and was derived from the application of pool samples consisting of 100 grains, with the goal to ensure the detection of 1 grain, which carries another genetic characteristic as the remaining pool.

#### Robustness

To assay the capacity of the method to remain unaffected by small variations, pipetting errors with increased (6 µL) as well as reduced (4 µL) DNA amounts were simulated. Additionally, the robustness of the method was measured by using reduced (4 µL) DNA quantity in combination with a reduced volume of reaction mixture (16 µL). Moreover, the temperature-time protocol was modified by raising the annealing temperature from 61 to 62 °C, and decreasing it from 61 to 60 °C.

#### Specificity

To exclude cross-reactivity with herbs, spices, nuts or cereals frequently used as food ingredients, 46 species listed in Table 3 were tested comprehensively. Therefore, DNA was extracted according to the *CTAB* protocol using the *Maxwell 16 FFS Nucleic Acid Extraction System Custom-Kit* (Promega, USA). For the subsequent PCR analysis, the extracts were diluted to an operating concentration of 5 ng/µL.

**Table 3:** Results of cross reactivity tests, obtained with 5 ng/µl DNA per tube.

Botanical family	Botanical name	Name		X̅ Ct-value of duplicate set-up^a^	∆Ct-value compared to hemp^b^
*Cannabaceae*	Cannabis sativa	Hemp		20.98	
	Humulus lupulus	Hops		35.46	14.48
*Moraceae*	*Morus*	Mulberry		38.06	17.61
	*Ficus*	Figs		39.24	18.26
*Urticaceae*	*Urtica*	Stinging nettle		43.26	22.28
*Asteraceae*	*Helianthus annuus*	Cornflower		40.56	19.58
*Vitaceae*	*Vitis*	Wine		41.77	20.79
*Lamiaceae*	*Lavandula*	Lavender		42.86	21.88
	*Rosmarinus officinalis*	Rosemary		-	-
	*Salvia*	Sage		-	-
	*Thymus*	Thyme		-	-
	*Satureja*	Savory		-	-
	*Origanum vulgare*	Oregano		-	-
*Fabaceae*	*Wisteria*	Wisteria		-	-
	*Vicia faba*	Beans		-	-
*Amaryllidaceae*	*Allium ampeloprasum*	Leek		-	-
*Apiaceae*	*Anethum graveolens*	Dill		-	-
	*Coriandrum sativum*	Coriander		-	-
	*Levisticum officinale*	Lovage		-	-
	*Petroselinum crispum*	Parsley		-	-
*Ulmaceae*	*Ulmus*	Elm		-	-
*Asteraceae*	*Artemisia dracunculus*	Tarragon		-	-
*Piperaceae*	*Piper nigrum*	Pepper		-	-
*Brassicaceae*	*Brassica nigra*	Black mustard		-	-
	*Brassica napus*	Rape		-	-
*Amaranthaceae*	*Amaranthus*	Amaranth			
	*Beta vulgaris*	Sugar beet		-	-
*Malvaceae*	*Gossypium*	Cotton		-	-
*Polygonaceae*	*Fagopyrum*	Buckwheat		-	-
*Anacardiaceae*	*Anacardium occidentale*	Cashew nuts		-	-
*Fagaceae*	*Castanea*	Chestnut		-	-
*Faboideae*	*Arachis hypogaea*	Peanuts		-	-
*Poaceae*	*Avena*	Oat		-	-
	*Triticum turgidum* × *polonicum*	Kamut		-	-
	*Zea mays*	Maize		-	-
	*Secale cereale*	Rye		-	-
	*Triticum*	Wheat		-	-
*Betulaceae*	*Corylus avellana*	Hazelnut		-	-
*Andropogoneae*	*Sorghum*	Millet		-	-
*Proteaceae*	*Macadamia*	Macadamia		-	-
*Rosaceae*	*Prunus dulcis*	Almond		-	-
*Lecythidaceae*	*Bertholletia excelsa*	Brazil nut		-	-
*Juglandaceae*	*Carya illinoinensis*	Pecan		-	-
	*Juglans regia*	Walnut		-	-
*Pedaliaceae*	*Sesamum indicum*	Sesame		-	-
*Zingiberaceae*	*Zingiber officinale*	Ginger		-	-
	*Solanum tuberosum*	Potato		-	-

- No increase of the fluorescence within 45 cycles

^a^ $$\stackrel{-}{\mathrm{X}}=\frac{{x}_{1}+{x}_{2}+\dots +{x}_{n}}{n}$$

^b^ $$\Delta \mathrm{Ct}-\mathrm{value }=\mathrm{ Ct}\, \mathrm{ crossreacting }\,\mathrm{ species }-\mathrm{ Ct }\,\mathrm{ hemp}.$$

#### Analysis of food samples

To verify the applicability of PCR analysis to identify hemp as a food ingredient, the real-time PCR assays were conducted with DNA extracts from diverse composed food products, with a DNA concentration of 5 ng/µL. The following food products were analysed with the developed real-time PCR method: Chocolate (milk, white, dark, (10–12% hemp)), cookies (9–15% hemp), pasta (5–12% hemp), muesli, tea, flour, patty (4% hemp), fruit bar (10% hemp), nibble hemp seeds and pesto (8% hemp).
